# Knowledge and attitude towards pediatric pain management among nurses at Ethiopian tertiary hospitals; a multi-center study

**DOI:** 10.1186/s12912-023-01234-8

**Published:** 2023-03-27

**Authors:** Takele Dereje Tagele, Yophtahe Woldegerima Berhe, Girmay Fitiwi Lema

**Affiliations:** 1 Department of Anesthesia, College of Medicine and Health Sciences, Aksum University, Aksum, Ethiopia; 2 Department of Anesthesia, College of Medicine and Health Sciences, University of Gondar, Gondar, Ethiopia

**Keywords:** Pain, Pediatric Pain, Pediatric Pain Management, Knowledge among nurses, Attitude among nurses

## Abstract

**Background:**

Pain is the most disturbing and annoying symptom experienced by children. However, it obtains poor attention in low- and middle-income countries particularly. The objective of this study was to assess knowledge, attitude and factors associated with pediatric pain management among nurses in tertiary hospitals in Northwest Ethiopia.

**Methodology:**

: A multi-center cross-sectional study was conducted from March 1 to April 30, 2021. The knowledge and attitude of nurses were measured by using Nurses’ Knowledge and Attitudes Survey regarding Pain (P-NKAS). Descriptive and binary logistic regression analyses were performed to determine factors associated with knowledge and attitude. The strength of the association was presented by using adjusted odds ratio with 95% confidence interval and p-value < 0.05 was considered as statistically significant.

**Result:**

A total of 234 (86.03% response rate) nurses were included and 67.1% of nurses had good knowledge and 89.3% had favorable attitudes towards pediatric pain management. The factors associated with good knowledge were having Bachelor’s Degree and above [AOR = 2.1, P = 0.015], having in-service training [AOR = 2.4, P = 0.008] and favorable attitude [AOR = 3.3, CI = 0.008]. The nurses who demonstrated good knowledge [AOR = 3.3, P = 0.003] and those who had Bachelor’s Degree and above [AOR = 2.8, P = 0.03] were found to have favorable attitude.

**Conclusion:**

The nurses who were working in pediatrics care areas had good knowledge and favorable attitude towards pediatrics pain management. However, improvements are needed to eradicate misconceptions; particularly, on pediatrics pain perception, opioid analgesia, multimodal analgesia, and non-pharmacologic pain therapies. Nurses who had higher level of education, in-service training, favorable attitude were found to be knowledgeable. Furthermore, nurses who had higher levels of education and knowledge were found to have favorable attitude.

## Introduction

Pain is the most disturbing and annoying symptom experienced by children. However, it obtains poor attention in low- and middle-income countries particularly [1–3]. Despite recent advancements in pain management, inadequate knowledge and unfavorable attitude of nurses remain a major barriers to provide effective pain management to children [4]. Personal values and beliefs of nurses about pain significantly affect pediatric pain management [5]. Poorly managed pain can result in multi-systemic adverse events and negatively affect the physical and psychological well-being of children, healthcare outcome, satisfaction and prolong hospital stay [3, 6]. Moreover, it also has direct and indirect healthcare cost implications; particularly in the low- and middle-income countries.

American Nurses’ Association claimed that the role of a nurse in pain management should include pain assessment, planning of pharmacologic and non-pharmacologic pain management strategies, implementing the plan, and evaluating the response of the patient to the interventions [7–9]. Despite these expected vital roles of nurses in pain management, multiple studies are revealing deficits in knowledge and attitude towards pain management [10–14]. Discrepant results were also reported on nurse’s knowledge and attitude towards pediatric pain management (15–61.1%) [8, 15, 16]. Pediatrics pain assessment and management have unique features and have been overlooked due to multiple factors such as limited resources, inadequate training and knowledge, unfavorable attitude, cultural diversity and language barriers [8, 9, 15–17]. There is limited research-based evidence regarding knowledge and attitude towards pediatric pain management among nurses working in low- and middle-income countries; especially in the sub-Saharan Africa. Therefore; this study aimed to assess the of knowledge, attitude and factors associated with pediatric pain management among nurses working in the tertiary hospitals in Northwest Ethiopia.

## Methodology

### Study design, area, population and period

**Fig. 1:**
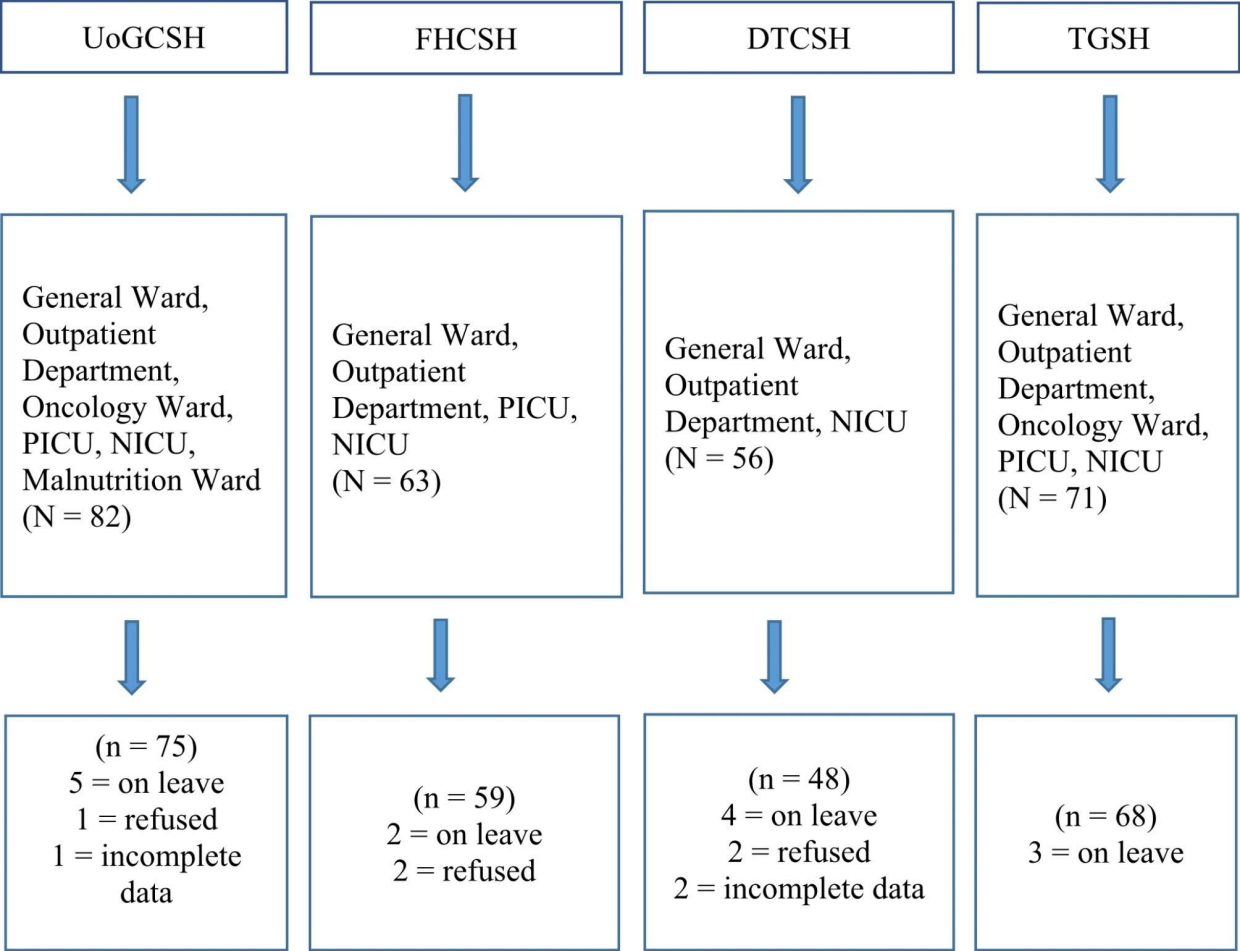
Nurses who were working in pediatric care areas in the tertiary hospitals in Northwest Ethiopia, 2021. NICU: Neonatal Intensive Care Unit, PICU: Pediatrics Intensive Care Unit

A multi-center cross-sectional census was conducted among nurses who were working at the four tertiary hospitals in Northwest Ethiopia. There were two tertiary university hospitals that are University of Gondar Comprehensive Specialized Hospital (UoGCSH) and Tibebe-Ghion Specialized Hospital (TGSH) and two tertiary governmental hospitals that are Felege-Hiwot Comprehensive Specialized Hospital (FHCSH) and Debre-Tabor Comprehensive Specialized Hospital (DTCSH). This study targeted to include all 272 nurses who had been working in different pediatric care areas in the hospitals from March 1 to April 30, 2021. Whereas, nurses who were on leave due to different reasons and refused to participate were excluded [Fig. 1].

#### Adequate knowledge

Knowledge was measured by using a tool developed by Miftah et al. Ten question items with total score ranging from 0 to 10 points were used to evaluate the of knowledge on pediatrics pain management among nurses and those who scored above the mean were considered to have good knowledge [8].

#### Inadequate knowledge

When scored below 8 to knowledge items [8].

#### Favorable attitude

Attitude was measured by using Nurses’ Knowledge and Attitudes Survey regarding Pain (P-NKAS). Twenty attitude items based on a 3-point rating scale ranging from 1 (disagree) to 3 (agree) with total score ranging from 20 to 60 points were used to evaluate nurses’ attitude towards pediatric pain management. Nurses who scored 50 and above were considered to have favorable attitude [18].

#### Unfavorable attitude

when scored below 50 to attitude items [18].

### Ethical approval, data collection and management

Ethical approval was obtained from the Ethical Review Committee of the School of Medicine, University of Gondar (**reference number: 479/04/2021**). Each participant has received adequate information about the study and informed consent was obtained. Confidentiality was ensured by locking data in secured area and removing identifiers. All methods were performed in accordance with the relevant guidelines and regulations.

Data were collected by using an adopted, structured, self-administered questionnaire which consists sociodemographic, work-related, knowledge, and attitude items in English language. The data collection instrument was declared valid and reliable by a previous study which calculated Kappa coefficient (0.78). Content validity was established through revision by experts skilled in pain management. Internal reliability for the instrument was 93% [18]. Data collection was conducted by 4 qualified anesthetists (one at each hospital). The data collectors were trained and supervised by the investigators. The collected data was checked, cleaned, and analyzed by using SPSS 20 (IBM Corporate).

Descriptive and inferential analyses were performed to determine the level and factors associated with knowledge and attitude towards pediatric pain management. Normality was tested by the Shapiro–Wilk test and the Hosmer and Lemeshow test was used to assess model fitness. The association of variables was determined by using binary logistic regression analysis. Bivariate analysis was performed and variables with p-value < 0.2 were fitted to the final multivariate analysis. The strength of associations between the independent variables and the dependent variables were presented in crude and adjusted odds ratio at a 95% confidence interval and. A p-value < 0.05 in the final model was considered as statistically significant.

## Results

**Fig. 2:**
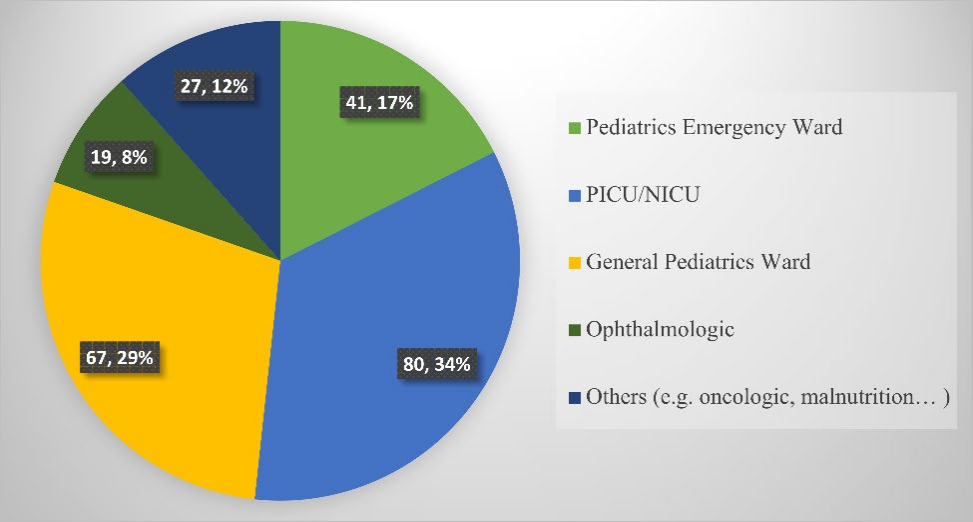
Nurses in pediatric care areas classified in working units NICU: Neonatal Intensive Care Unit, PICU: Pediatric Intensive Care Units

A total of 272 eligible participants were approached and data obtained from 234 nurses (86.0% response rate) was used for final analysis. The majority of the participants (126 (53.8%)) were males. The ages of the participants range from 22 to 50 and the mean ± standard deviation of age was 29.5 ± 4.4 years. Almost near to half of the participants 104 (44.4%) had 3–5 years of work of experience [Table 1]. Most of the nurses 80 (34.2%) were working in either of pediatric intensive care units (PICU) or neonatal intensive care units (NICU) [Fig. 2].

**Table 1:** Characteristics of nurses who were working in pediatric care in tertiary hospitals in Northwest Ethiopia, 2021, N = 234

Variables	Frequency	Percentage
Sex		
Male Female	126 108	53.8 46.2
Work experience (years)		
≤ 2 3–5 ≥ 5	46 104 84	19.7 44.4 35.9
Levels of education		
Diploma Bachelor’s Degree and above	19 215	8.1 91.9
Training on pain management		
Yes No	89 149	38.0 62.0

The mean ± SD of knowledge scores was 8.13 ± 1.7 and the results range from 1 to 10. More than 2/3rd (67.1%) of the nurses were found to have good knowledge (CI = 61.1–73.5) and 32.9% of the nurses were found to have poor knowledge (CI = 26.5–38.9). Out of 234 respondents, 80.3% correctly answered that increasing analgesics of narcotics is sign of addiction and 85.0% correctly answered that distraction of pain by music and relaxation is appropriate to manage pain among children [Table 2].

**Table 2:** Evaluation of the level of knowledge of nurses on pediatric pain management

No.	Questions	Responses	Frequency	Percentage
1.	Narcotics on a regular schedule is preferred over PRN schedule for continuous pain	Yes No	160 74	68.4 31.6
2.	Accurate judge of the intensity of the patient’s pain is the patient	Yes No	175 59	74.8 25.2
3.	Distraction by use of music or relaxation decrease feeling of pain	Yes No	199 35	85 15
4.	Increasing narcotic analgesic requirement are signs, patient is becoming addicted	Yes No	188 46	80.3 19.7
5.	Severe chronic pain often needs higher dosages of pain medications than acute pain	Yes No	192 42	82.1 17.9
6.	Narcotics for pediatric patients can cause respiratory depression	Yes No	187 47	79.9 20.1
7.	Analgesics for chronic pain are needed	Yes No	206 28	88 12
8.	Analgesics for cancer pain are needed	Yes No	208 26	88.9 11.1
9.	Reports of patient/family, narcotic causing euphoria, should be given a lower dose of the analgesic	Yes No	168 66	71.8 28.2
10.	Do children need better attention for managing their pain?	Yes No	215 19	93.6 6.4

In the bivariate binary logistic regression analysis; sex, level of education, work experience, having in-service training on pain management, and attitude towards pain management were found to be fit for the final analysis (P < 0.2). The final multivariate analysis showed that level of education, having in-service training on pain management, attitude towards pediatric pain management were found independently associated with knowledge on pediatric pain management among nurses (P < 0.05).

Compared to nurses who had diploma qualification, those who had Bachelor’s degree and above were two times more knowledgeable [AOR = 2.1, CI = 1.1–5.7, P = 0.015]. Nurses who had in-service training on pain management were more than twice knowledgeable than those who had not [AOR = 2.4, CI = 1.3–4.8, P = 0.008]. Furthermore, nurses who had favorable attitude towards pediatric pain management were about three times knowledgeable compared to nurses who had unfavorable attitude [AOR = 3.3, CI = 1.4–8.1, P = 0.008] (Table 3).

**Table 3:** Binary logistic regression analysis: factors associated with knowledge of nurses on pediatric pain management

Variables	Poor Knowledge n (%)	Good Knowledge n (%)	Odds Ratio (95% CI)		P-value
			Crude	Adjusted	
Sex					
Male Female	46 (36.5) 31 (28.7)	80 (63.5) 77 (71.3)	0.7 (0.4–1.2) 1	0.7 (0.4–1.2) 1	0.17
Levels of education					
Diploma Bachelor’s Degree and above	10 (52.6) 67 (31.2)	9 (47.4) 148 (68.8)	1 2.5 (1.4–6.3)	1 2.1 (1.1–5.7)	1 **0.015**
Work experience (years)					
≤ 2 3–5 ≥ 5	18 (39.1) 28 (26.9) 31 (36.9)	28 (60.9) 76 (73.1) 52 (63.1)	1 1.7 (0.8–3.6) 1.1 (1.0–2.3)	1 1.7 (0.8–3.7) 0.9 (0.4–2.0)	1 0.17 0.82
In-service training on pain management					
Yes No	21 (23.6) 56 (38.6)	68 (76.4) 89 (61.4)	2.0 (1.1–3.7) 1	2.4 (1.3–4.8) 1	**0.008** 1
Attitude towards pain management					
Unfavorable Favorable	15 (60.0) 62 (29.7)	10 (40.0) 147 (70.3)	1 3.6 (1.5–8.3)	1 3.3 (1.4–8.1)	1 **0.008**

The attitude scores range from 38 to 59 and the median (IQR) was 54 (51–55). The majority of nurses (209 (89.3%)) were found to have favorable attitude towards pediatric pain management. Nurses were asked to answer agree/disagree/not sure to items that were prepared to assess their attitude. Around 95.3% of nurses agreed that measurement and control of pain in a child leads to improved quality of child’s life and over 90% believe that pain should be considered as a vital sign and assessed and documented during examination. However, only 17.1% of nurses believed that children experience pain as much as adults though 23.9% were not sure. 59% (59%) of the nurses did not believe that children experience pain equal to that experienced by adults. About 86.3% of nurses stated that infants and children have the right to be appropriately assessed and managed for their pain. About 46.2% of nurses agreed that when the necessary procedures have been done for the patient, the persistence of pain does not cause problems and 4.3% of nurses disagreed that failure to assess and manage children’s pain affects the body and mind in the long term. About 43.6% of nurses believed that parents should be present during painful procedures while the remaining were not sure (29.9%) and disagreed (26.5%). About 73.8% of nurses agreed that the most accurate judge of the intensity of the children’s pain is the primary nurse and 73.9% approved the significance of assessment tools for effective pain measurement and appropriate pain management (Table 4).

**Table 4:** Evaluation of attitude of nurses towards pediatric pain management

No.	Question items	Agree n (%)	Not sure n (%)	Disagree n (%)
1.	Infants and children experience pain equal to that experienced by adults	40 (17.1)	56 (23.9)	138 (59.0)
2.	Parents should be present during painful procedures	102 (43.6)	70 (29.9)	62 (26.5)
3.	Pain management and pain relief are of priority in children treatment	194 (82.9)	38 (16.2)	2 (0.9)
4.	Children have the right to appropriate assessment and management of their pain	202 (86.3)	27 (11.5)	5 (2.1)
5.	The most accurate judge of the intensity of the children’s pain is the her/his primary nurse	172 (73.8)	47 (20.1)	15 (6.4)
6.	Full treatment of pain is a humanitarian issue	191 (81.6)	37 (15.8)	6 (2.6)
7.	To better assess child pain, the nurse can discuss with her/his parents	197 (84.2)	33 (14.1)	4 (1.7)
8.	Assessment and control of child pain can lead to improved his/her parent’s satisfaction	198 (84.6)	28 (12)	8 (3.4)
9.	Failure to assess and manage the child’s pain affects his body and mind in the long term	188 (80.3)	36 (15.4)	10 (4.3)
10.	The nurse’s physical and mental fatigue can affect children pain relief	196 (83.8)	25 (10.7)	13 (5.6)
11.	Like other vital signs, pain score should be documented	215 (91.9)	8 (3.4)	11 (4.7)
12.	To ensure patient’s comfort and pain relief is one of the most important tasks of nurses	195 (83.3)	34 (14.5)	5 (2.1)
13.	Communicating with and educating child’s parents play an effective role in relieving pain	199 (85.0)	31 (13.2)	4 (1.7)
14.	Available tools for measurement of pain are the best for determining pain severity in children	196 (83.8)	31 (13.2)	7 (3.0)
15.	When the necessary procedures have been done for the patient, the persistence of pain does not cause problems	108 (46.2)	64 (27.4)	62 (26.5)
16.	Using pain assessment tool for determining child’s pain leads to an appropriate method of pain relief	173 (73.9)	46 (19.7)	15 (6.4)
17.	Measurement and control of child’s pain can affect the healing process and reduces the hospital	174 (74.4)	37 (15.8)	23 (9.8)
18.	Evaluation and measurement of child’s pain should be considered as one of the vital signs when examining the child	216 (92.3)	15 (6.4)	3 (1.3)
19.	Comparable stimuli in different people produce the same intensity of pain	153 (65.4)	39 (16.7)	42 (17.9)
20.	Measurement and control of pain in child leads to improved quality of child’s life	223 (95.3)	8 (3.4)	3 (1.3)

In the final multivariate analysis, higher levels of education and knowledge on pediatric pain management were found independently associated with nurses’ attitude towards pediatrics pain management (P < 0.05). Nurses who had good knowledge on pediatric pain management were over three times more likely to have favorable attitude towards pediatric pain management than those who had poor knowledge [AOR = 3.3, CI = 1.4–7.8, P = 0.007]. Furthermore, having a Bachelor’s Degree and above increased the likelihood of having a favorable attitude towards pediatric pain management by nearly three times [AOR = 2.8, CI = 1.1–9.8, P = 0.03] (Table 5).

**Table 5:** Binary logistic regression analysis: factors associated with attitude of nurses towards pediatric pain management

Variables	Attitude towards pediatric pain management		Odds ratio (95% CI)		P-value
	Unfavorable n (%)	Favorable n (%)	Crude	Adjusted	
Sex					
Male Female	13 (10.3) 12 (11.1)	113 (89.3) 96 (88.9)	1.1 (0.4–2.5) 1	*	*
Levels of education					
Diploma Bachelor’s Degree and above	5 (26.3) 20 (9.3)	14 (73.7) 195 (90.7)	1 3.5 (1.1–10.7)	1 2.8 (1.1–9.8)	**0.03**
Work experience (years)					
≤ 2 3–5 ≥ 5	4 (8.7) 10 (9.6) 11 (13.1)	42 (91.3) 64 (90.4) 73 (86.9)	*	*	*
In-service training on pain management					
Yes No	9 (10.1) 16 (11.0)	80 (89.9) 129 (89.0)	1 1.1 (0.5–2.6)	*	*
Level of knowledge					
Good knowledge Poor knowledge	10 (6.4) 15 (19.5)	147 (93.6) 62 (80.5)	3.6 (1.5–8.3) 1	3.3 (1.4–7.8) 1	**0.007** 1

## Discussion

Pain is one of the commonest complaints presented to nurses and they are expected to have adequate knowledge and favorable attitude towards pain management [7, 15]. In the current study, good knowledge was observed among 67.1% of nurses which is comparable to previous studies that reported 60.0%, 61.1%, and 66.8% of nurses had good knowledge [11, 16, 19]. However, it is relatively higher than previous findings which reported 41.0%, 48.2% and 58.6% of nurses had good knowledge [8, 18, 20].

Moreover, we have observed favorable attitude towards pediatric pain management among 209 (89.3%) nurses and the median (IQR) of attitude scores was 54 (51–55) which is comparable to previous studies [17, 18]. However, these results are superior compared to previous studies in which over the half of nurses found to have unfavorable attitude [4, 12, 13]. The differences might be explained as due to large proportion of nurses (91.1%) who held Bachelor’s Degree or above were included in our study. Inclusion of nurses non-specific to pediatric care areas in the previous studies and utilization of different assessment tools might also explain the discrepancies.

The majority of nurses (93.6%) responded that children need better attention for managing their pain; 92.3% believed that pain assessment must be considered as vital sign examination and 81.6% agreed that full treatment of pain is a humanitarian issue. However, only 17.1% of nurses correctly answered that infants and children experience pain equal to that experienced by adults which is consistent to a previous study [21]. Moreover, 46.2% of nurses agreed to the statement “when the necessary procedures have been done for the patient, the persistence of pain does not cause problems.” In previous misconceptions, it was thought that infants did not feel pain because of an immature nervous system. Neonates and infants feel pain; even, react to noxious stimuli with distress indicative of pain [22].

Seventy-four (31.6%) nurses believed that narcotics on regular schedules are not preferred options for continuous pain. Consistently, a previous study found out 37.8% of nurses believed that patients should be encouraged to endure as much pain as possible before using an opioid [23]. Furthermore, another study also reported low rate of correct answers (30.8%) for the questions regarding opioids [14]. Trends of overestimation of opioids as dangerous drugs made clinician reluctant to use them. Side-effects and abuse of opioids has limited their use and led to inadequate information and attitudes towards opioids. Provision of training focusing on opioid use may enhance correctness [23].

Regarding use of non-pharmacological approaches of pain management, about 85.0% of nurses confirmed that distraction by use of music or relaxation decrease feeling of pain. A previous study found that 60.5% of nurses incorrectly believe that if a child can be distracted from pain it is not severe. 79% erroneously believe that children with severe pain will not be able to sleep. Effective use of non-pharmacological approaches needs better understanding of childhood behavior and pain responses [4].

About 74.8% believed that the patient is the accurate judge of the intensity of pain. However, previous studies showed that more than half of nurses incorrectly answered the item ‘‘The most accurate judge of the intensity of the child’s pain is the child’’ suggesting that a child’s self-reported pain is not trusted by nurses as reliable during assessment practices [4, 15]. Evidences suggested that self-reported tools can be reliably used by children as young as three years [24, 25].

Our study revealed that having Bachelor’s Degree or above in Nursing was associated with both good knowledge and favorable attitude compared to have Diploma in Nursing. When nurses have a minimum of Bachelor’s Degree in Nursing, the odds of having good knowledge and favorable attitude were double [AOR = 2.1, P = 0.015] and triple times higher [AOR = 2.8, P = 0.03] respectively. Consistently, previous studies declared that higher educational level was associated with good knowledge and favorable attitude towards pain management among nurses [6, 15, 18, 23, 26]. However, there are previous studies that stated no significant association between level of education and knowledge and attitude [12, 13]. This finding might be explained by bigger contents concerning pain management in the curricula for higher Degree programs (Bachelor’s and Master’s) compared to that of Diploma programs.

Nurses who have had in-service training on pain management were found to be knowledgeable more than twice [AOR = 2.4, P = 0.008]. Previous studies have reported that lack of training resulted in inadequate knowledge and affected the provision of pain management. In-contrast, education and training on pain management enhanced knowledge and practice of pain management [23, 27]. However, a recent study has reported no significant differences were identified for the exposure to previous pain education and explained it by the shortage of quality continuing medical education courses on topics of pain management [12].

Knowledge and attitude are inter-related entities that influence one another. In the current study, nurses who had favorable attitude were found over triple times more knowledgeable [AOR = 3.3, P = 0.008]. In-reverse, nurses who had good knowledge were found to have favorable attitude [AOR = 3.3, P = 0.007]. Similarly, higher knowledge was correlated to more positive attitude towards pain management and suggested that knowledge levels need to be improved to ensure more positive attitude to pain management [22]. However, there are studies that showed knowledge and attitude can be independent of one another suggesting nurses may have favorable attitude towards pain management with inadequate knowledge [17, 19].

Our findings denied the associations of nurses’ age, sex, work experience, and units with knowledge and attitude towards pediatric pain management which is consistent to some previous studies [13, 16, 23, 26]. Even though, a previous study has found significantly higher mean score of knowledge and attitude among female nurses [12].

The limitations of this study are; due to its cross-sectional design, it could not draw temporal relations; and it does not have qualitative components that might enable to explore barriers and root-causes for ineffective pediatric pain management. Furthermore, the study was conducted only in the tertiary hospitals in the region. It did not include lower healthcare facilities that could lack resources and expertise for pediatric pain management. We expect more exaggerated problems in pediatric pain management in those facilities.

## Conclusion

The nurses who were working in pediatric care areas had good knowledge and favorable attitude towards pediatric pain management. However, improvements are still needed to abolish misconceptions; particularly, on pediatric pain perception, opioid analgesia, multimodal analgesia, and non-pharmacologic pain therapies. Nurses who had higher level of education, in-service training, favorable attitude were found to be knowledgeable. Furthermore, nurses who had higher levels of education and knowledge were found to have favorable attitude. According to these premises, we urge the hospitals to deploy nurses who have higher level of education at pediatricscare areas, and provide quality pre-service educational and regular in-service training opportunities to enhance knowledge and attitude towards pediatric pain management among nurses.

## Data Availability

Data and materials used in this study are available and can be presented by the corresponding author upon reasonable request.

## Abbreviations

AOR: Adjusted Odd Ratio
COR: Crude Odd Ratio
DTCSH: Debre Tabor Comprehensive Specialized Hospital
FHCSH: Felege-Hiwot Comprehensive Specialized Hospital
IQR: Inter-quartile Range
NICU: Neonatal Intensive Care Unit
PICU: Pediatric Intensive Care Unit
PNKAS: Nurses’ Knowledge and Attitudes Survey regarding Pain
SD: Standard Deviation
SPSS: Statistical Package for the Social Sciences
TGSH: Tibebe-Ghion Specialized Hospital
UoGCSH: University of Gondar Comprehensive Specialized Hospital
